# Spatial–Temporal Variation of Grain Magnesium, Calcium and Their Response to Phosphorus Nutrition in Sweet Corn

**DOI:** 10.1002/fsn3.4725

**Published:** 2025-01-25

**Authors:** Da Su, Zhiya Jin, Jie Ou, Muhammad Atif Muneer, Yunfei Jiang, Delian Ye, Liangquan Wu, Xiaojun Yan

**Affiliations:** ^1^ Key Laboratory of Genetics, Breeding and Multiple Utilization of Crops, Ministry of Education; Key Laboratory of Biological Breeding for Fujian and Taiwan Crops, Ministry of Agriculture and Rural Affairs, College of Agriculture Fujian Agriculture and Forestry University Fuzhou China; ^2^ International Magnesium Institute, College of Resources and Environment Fujian Agriculture and Forestry University Fuzhou China; ^3^ Department of Plant, Food, and Environmental Sciences, Faculty of Agriculture Dalhousie University Truro Nova Scotia Canada

**Keywords:** biofortification, phosphorus, phytic acid, sweet corn ( *Zea mays* L. *saccharata*)

## Abstract

Sweet corn ( *Zea mays* L. *saccharata*) is gaining global popularity as a staple crop and a vegetable due to its high nutritional value. However, information on grain magnesium (Mg) and calcium (Ca) status and their response to phosphorus (P) fertilization in sweet corn is still insufficient. In the current study, we combined the genotype evaluation and P gradient experiments to investigate how P supply influences the spatio‐temporal variation of grain mineral nutrition (Mg and Ca) and their bioavailabilities in sweet corn. Increasing P decreased grain Ca by 16.7%, from 0.18 to 0.15 g kg^−1^, but increased phytic acid phosphorus (PAP) concentration by 7.4%. Optimal P levels increased grain Mg concentration by 14.7%, from 1.36 to a peak of 1.56 g kg^−1^; however, excessive P reduced it by 3.8% to 1.50 g kg^−1^. Spatially, optimal P increased the grain Mg concentration across all cob positions and in the germ, with the largest variation observed at the upside position (Coefficient of variation (cv) = 11.88%). Conversely, high P decreased Mg concentration in middle‐cob grains and the germ. High P also reduced Ca in the upper/middle‐cob grain and in both germ and endosperm. P consistently increased grain PAP but reduced Mg and Ca bioavailability across all growth stages, all grain fractions, and the middle/bottom cob positions. Notably, PAP showed the largest variation at the late growth stage in bottom cob grains (CV = 8.75%). Mg and Ca bioavailabilities exhibited significant variations, primarily in upper grains during the early filling stage, with CVs reaching 14.5% and 43.23%, respectively. Temporally, early grain growth stage was more sensitive to P‐induced changes in Mg, Ca and their bioavailabilities, while later growth stage was more responsive to grain PAP alterations. These findings suggest that reducing P input can be advantageous for achieving high Mg and Ca biofortified sweet corn grain.

## Graphical abstract

High P reduced grain Mg, Ca bioavailability in all grain stages, fractions. Phytate in pericarp, Ca/Mg bioavailability in endosperm/germ, highly sensitive to P. Rational reduction of P is beneficial to obtain Mg/Ca biofortified sweet corn grain.

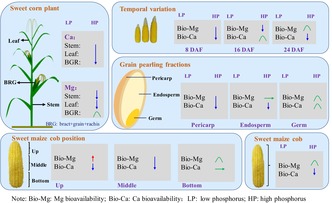

**Abbreviations**
BGR: bract + grain + rachis
Ca: calcium
CK: no phosphorus fertilizer used
CV: coefficient of variation
DAP: days after pollination
HP: high phosphorus levels
LP: low phosphorus level
Mg: magnesium
P: phosphorus
PA: phytic acid
[PA]/[Ca]: the molar ratio of phytic acid to calcium
[PA]/[Mg]: the molar ratio of phytic acid to magnesium
PAP: phytic acid phosphorus

## 1 Introduction

Sweet corn ( *Zea mays* L. *saccharata*) is a distinctive vegetable crop due to its exceptionally high sweetness in the grain's endosperm. This exceptional sweetness is attributed to mutations of the recessive shrunken2 (*sh2*) gene located on chromosome 3 L (Lertrat and Pulam 2007; Revilla et al. 2006). These mutations hinder the conversion of sugar to starch, leading to the accumulation of higher levels of sucrose and other soluble sugars in the endosperm. In recent years, sweet corn has gained significant acceptance among consumers primarily due to its desirable palatability, superior organoleptic properties, and higher antioxidant activity and phytochemical content even after processing (Lertrat and Pulam 2007; Revilla et al. 2006). In addition to its sweet grains and consumer appeal, sweet corn byproducts, such as sweet corn cob selenium polysaccharide (SeSCP), also hold potential for functional food and medicinal applications by improving intestinal barrier function, reducing inflammation, regulating gut microbiota, and enhancing carbohydrate and lipid metabolism (Wang et al. 2024; Xiu et al. 2024).

China has emerged as the second‐largest sweet corn producer , contributing nearly a quarter of global production from its planting area (Wang et al. 2021). Southern subtropical provinces, such as Guangdong and Fujian, are the major regions for sweet corn production. These regions possess ample thermal and precipitation resources, enabling multiple cropping throughout the year. However, despite the productivity advantages, challenges persist in the form of widespread soil magnesium (Mg) and calcium (Ca) deficiencies in the acidic soils of these regions (Wang et al. 2022). Moreover, the long‐term excessive use of chemical fertilizers, particularly in intensive sweet corn farming practices, reduces soil pH, intensifying soil mineral deficiencies, especially Mg (Chen et al. 2022). Among these chemical fertilizers, the overuse of phosphorus (P) fertilizer, a valuable nonrenewable resource, has emerged as a major challenge. Yan et al. (2022) explored the spatial variation of soil P in subtropical regions of southern China and found that soil available P had increased by approximately 42 times over the past 30 years.

Studies have extensively investigated the impact of P fertilizer on crop yield potential and P‐use efficiency (Wu et al. 2015; Yan et al. 2021). P fertilization strategies influence grain quality as well. Overuse of P has been reported to adversely affect taste value and eating quality (Chen et al. 2023). Additionally, meta‐analyses have concluded that high P (HP) reduces the quantity of zinc (Zn) and iron (Fe) in many plant species (Zhang et al. 2017, 2021). In addition to Zn and Fe, grain Mg and Ca are essential for human health. Mg, the fourth most abundant element in the human body, is vital for various physiological functions. Epidemiological statistics have shown that the daily allowance of Mg is generally insufficient in most populations, leading to an increased risk of various clinical manifestations (Pelczyńska, Moszak, and Bogdański 2022). Dietary Ca deficiency is another significant global concern, affecting nearly half of the global population (Wang et al. 2023). The grains of maize, a major staple crop, are rich in dietary fiber and vitamins such as provitamin A and vitamin E, and they offer higher niacin accessibility when cooked (Nuss and Tanumihardjo 2010). However, they have been found to have inadequate or poorly balanced level of essential mineral nutrients, such as Mg, Ca, Zn, and Fe (Nuss and Tanumihardjo 2010; White and Broadley 2005). This creates a solid gap in achieving the biofortification target of mineral values. Among the minerals, limited research has been concentrated on biofortification strategies for Mg and Ca and their bioavailabilities. Therefore, exploring possible P‐induced alteration in grain mineral concentrations beyond Zn and Fe, especially in less‐reported sweet corn on acidic and Mg/Ca‐deficient soils in southern China, is essential to comprehensively understand the influence of P fertilization on plant mineral nutrition (Wang et al. 2022).

In the present study, we used different sweet corn cultivars and P gradient experiments to explore the impact of P fertilizer on the mineral nutrition of sweet corn grains. The objectives of this study were (1) to investigate the temporal and spatial variations, specifically in terms of cob position and grain fractions of grain phytic acid phosphorus (PAP), Mg, and Ca concentrations and mineral bioavailabilities in sweet corn; (2) to investigate the influence of P supply on the grain PAP, Mg, and Ca concentrations as well as mineral bioavailabilities from temporal and spatial distribution perspectives. The findings could provide useful information for the biofortification of Mg and Ca in sweet corn through optimized P management strategies.

## 2 Materials and Methods

### 2.1 Experimental Area

The experiment was conducted at the sweet corn experimental farm in Zhangzhou City, Fujian Province (E 117.22°, N 23.72°). This region has a typical subtropical monsoon climate with sufficient thermal and precipitation resources. The soil type was red soil, and the basic soil characteristics are shown in Table S1.

### 2.2 Experimental Design

This study conducted two experiments: (1) the different sweet corn cultivars and (2) the P gradient experiment.

#### 2.2.1 Experiment 1: The Different Sweet Corn Cultivar Experiment (2018)

The sweet corn cultivar experiment aimed to understand the impact of P on Mg and Ca concentrations in grain, as well as their bioavailabilities, among different sweet corn genotypes. A split‐plot design experiment with three replications was conducted. Four sweet corn genotypes (e.g., Guangliangtian No. 31, Minshuangse No. 4, Xiantian No. 5, and Yongzhen No. 7) were the main plot, and two P fertilizer levels (CK with no P used and HP with 120 kg P_2_O_5_ ha^−1^) were split‐plot. All genotypes were commercial hybrids cultivated under standard field management practices. Sowing took place in mid‐September, and harvesting occurred in mid‐December.

#### 2.2.2 Experiment 2: Gradient Experiment (2019)

A P gradient experiment was further performed to explore the spatio‐temporal variation of grain minerals (Mg and Ca) and their bioavailabilities and assess their response to P fertilization under different P levels. For this experiment, the Xiantian No. 5 genotype was selected from experiment 1 because it is one of the most locally planted sweet corn genotypes and most susceptible to P fertilizer for grain PAP concentration. This experiment was performed under a completely randomized block design with four P application rates: P_0_ (0 kg P_2_O_5_ ha^−1^, no P supply), P_1_ (75 kg P_2_O_5_ ha^−1^, low P level), P_2_ (150 kg P_2_O_5_ ha^−1^, medium P level), and P_3_ (300 kg P_2_O_5_ ha^−1^, HP level). Xiantian No. 5 was sown in the middle of September and harvested in the middle of December.

For both field experiments, sweet corn seedlings were grown in trays and then transplanted into the field. The entire growth period followed local, high‐yield field management practices. Fertilizer application was as follows: P fertilizer was applied as a basal dressing using the spot application method. Apart from P fertilizer, nitrogen (N) and potassium (K) were also applied at consistent rates. The N fertilizer application rate was 200 kg N ha^−1^, divided into three applications (basal dressing: during the elongation stage: during the silk stage = 3:4:3). The K fertilizer application rate was 120 kg K_2_O ha^−1^, divided into two applications (basal dressing: during the silk stage = 5:5).

### 2.3 Sampling

For Experiment 1, at the harvest stage, the grains of representative sweet corn plants with consistent plant growth, height, and similar cob shape and height of four genotypes were selected to analyze the concentration of grain PAP, Mg, Ca, and the molar ratio of phytic acid (PA) to minerals. For Experiment 2, since local sweet corn was typically harvested 20–25 days after pollination, grains were collected at 8, 16, and 24 days after pollination (DAP), representing the early, middle, and prime harvest phases, respectively. The samples of different grain growth stages were further separated based on the grain position in the cob, namely, the upside, middle, and bottom positions of the cob (developing grains from the tip to the base were evenly divided into three parts of the sweet corn cob). Moreover, at harvest time intact sweet corn grains from the middle position of the cob were dissected into three distinct fractions: pericarp (seed coat), endosperm, and germ. The representative sweet corn plants of Xiantian No. 5 were separated into stem, leaf, and bract + grain + rachis (BGR).

Samples, which included both grain and vegetative tissues, were separately collected from each replication. After undergoing a drying process in an oven until a constant weight was reached, the grains were ground into powder with a mortar and pestle to avoid potential metal contamination from using a grinder. The vegetative tissues were cut and milled using a portable grinder. All milled powders were subsequently passed through a 0.25‐mm sieve to achieve a fine, homogeneous powder. These samples were then stored in a thick polyethylene bag in the refrigerator. All treatments were analyzed using the mean values of three replicates.

### 2.4 Chemical Analyses

Grain PA concentration was determined with the precipitation of ferric phytate, as described by Su et al. (2018). The grain PAP was calculated by multiplying PA by 0.2818. Grain Mg and Ca concentrations on a dry weight basis (g kg^−1^) were measured using inductively coupled plasma optical emission spectroscopy (ICP‐OES, OPTIMA 3300 DV, Perkin‐Elmer, USA), following the method described by Zhang et al. (2017). The bioavailability of Mg and Ca was estimated using the molar ratio of PA to Mg and Ca as follows:

$$\text{The molar ratio of}\;\mathrm{PA}\;\text{to}\;\mathrm{Ca}=\frac{\mathrm{PA}\;\left(\mathrm{mg}\;100g^{-1}\right)/660}{\mathrm{Ca}\;\left(\mathrm{mg}\;100g^{-1}\right)/40.078}$$

$$\text{The molar ratio of}\;\mathrm{PA}\;\text{to}\;\mathrm{Mg}=\frac{\mathrm{PA}\;\left(\mathrm{mg}\;100g^{-1}\right)/660}{\mathrm{Mg}\;\left(\mathrm{mg}\;100g^{-1}\right)/24.31}$$

### 2.5 Statistical Analysis

The results were expressed as the mean ± standard deviation. Data were subjected to analysis of variance using IBM SPSS Statistics 21, and the differences between means were compared by Tukey's multiple comparison test at *p* < 0.05 (*). Bar charts were plotted using GraphPad Prism 8 (San Diego, CA, USA). A parallel plot was drawn using OriginPro 2019b.

## 3 Results

### 3.1 Effect of P Fertilizer on Grain Mg and Ca Concentrations and Their Bioavailabilities Across Different Sweet Corn Genotypes

P fertilization influenced not only the concentration (on a dry matter basis) of grain Mg and Ca but also their bioavailabilities ([PA]/[Mg] and [PA]/[Ca], Table 1). Grain Ca concentrations significantly decreased with P supply in most sweet corn genotypes (except for Guangliangtian No. 31). In contrast, grain PAP concentrations increased significantly with P fertilization across all genotypes. The P‐induced variation in grain Mg concentration was genotype‐dependent. Two genotypes (Guangliangtian No. 31 and Minshuangse No. 4) increased grain Mg concentration with P application. The other two genotypes (Xiantian No. 5 and Yongzhen No. 7) showed the opposite trend. Influenced by the P‐induced variations of PAP and mineral (Mg and Ca) concentrations, [PA]/[Mg] was remarkably increased (Xiantian No. 5 and Yongzhen No. 7) or remained stable (Guangliangtian No. 31 and Minshuangse No. 4). By contrast, [PA]/[Ca] of all sweet corn genotypes was significantly increased with P application.

**TABLE 1:** Effect of phosphorus (P) fertilizer application on magnesium (Mg) and calcium (Ca) concentrations and their estimated bioavailabilities in sweet corn grain across different genotypes.

Genotype	Treatment	PAP (g kg^−1^)	Mg (g kg^−1^)	Ca (g kg^−1^)	[PA]/[Mg]	[PA]/[Ca]
Guangliangtian No.31	CK (g kg^−1^)	2.08 ± 0.04	1.31 ± 0.01	0.22 ± 0.01	0.21 ± 0.00	2.04 ± 0.10
	HP (g kg^−1^)	2.27 ± 0.06	1.36 ± 0.02	0.2 ± 0.02	0.22 ± 0.01	2.5 ± 0.22
	D‐value (g kg^−1^)	0.19	0.05	−0.02	0.01	0.46
	Fluctuating rate (%)	0.09**	0.04*	−0.11^ns^	0.03^ns^	0.23*
Minshuangse No.4	CK (g kg^−1^)	2.22 ± 0.00	1.17 ± 0.01	0.15 ± 0.01	0.25 ± 0.00	3.12 ± 0.11
	HP (g kg^−1^)	2.34 ± 0.02	1.25 ± 0.02	0.13 ± 0.01	0.24 ± 0.01	3.78 ± 0.12
	D‐value (g kg^−1^)	0.12	0.09	−0.02	−0.01	0.66
	Fluctuating rate (%)	0.06*	0.07**	−0.13*	−0.03^ns^	0.21**
Xiantian No.5	CK (g kg^−1^)	2.11 ± 0.05	1.31 ± 0.00	0.17 ± 0.01	0.21 ± 0.00a	2.63 ± 0.20
	HP (g kg^−1^)	2.26 ± 0.03	1.29 ± 0.01	0.15 ± 0.01	0.23 ± 0.00a	3.31 ± 0.14
	D‐value (g kg^−1^)	0.15	−0.02	−0.03	0.02	0.68
	Fluctuating rate (%)	0.07**	−0.02*	−0.15*	0.08**	0.26**
Yongzhen No.7	CK (g kg^−1^)	2.21 ± 0.03	1.29 ± 0.00	0.23 ± 0.01	0.22 ± 0.01	2.1 ± 0.07
	HP (g kg^−1^)	2.28 ± 0.01	1.26 ± 0.01	0.15 ± 0.00	0.24 ± 0.00	3.28 ± 0.02
	D‐value (g kg^−1^)	0.08	−0.03	−0.08	0.02	1.18
	Fluctuating rate (%)	3.47**	−2.33**	−33.82**	7.46**	56.03***

*Note:* CK: no phosphorus fertilizer used; HP: relative high phosphorus fertilizer treatment (120 kg P_2_O_5_ ha^−1^). The values presented are the means of three replications. The symbols *, **, *** and ns, used for a specific sweet corn genotype under two different phosphorus fertilizer treatments, indicate significant differences at *p* < 0.05, *p* < 0.01, *p* < 0.001, and no significant difference, respectively.

Abbreviations: PA, phytic acid; PAP, phytic acid phosphorus; [PA]/[Mg], the molar ratio of phytic acid to magnesium; [PA]/[Ca], the molar ratio of phytic acid to calcium.

### 3.2 Response of P Application on Plant Biomass and Distribution of P, Mg, and Ca Concentrations in Above‐Ground Plant Organs

**FIGURE 1:**
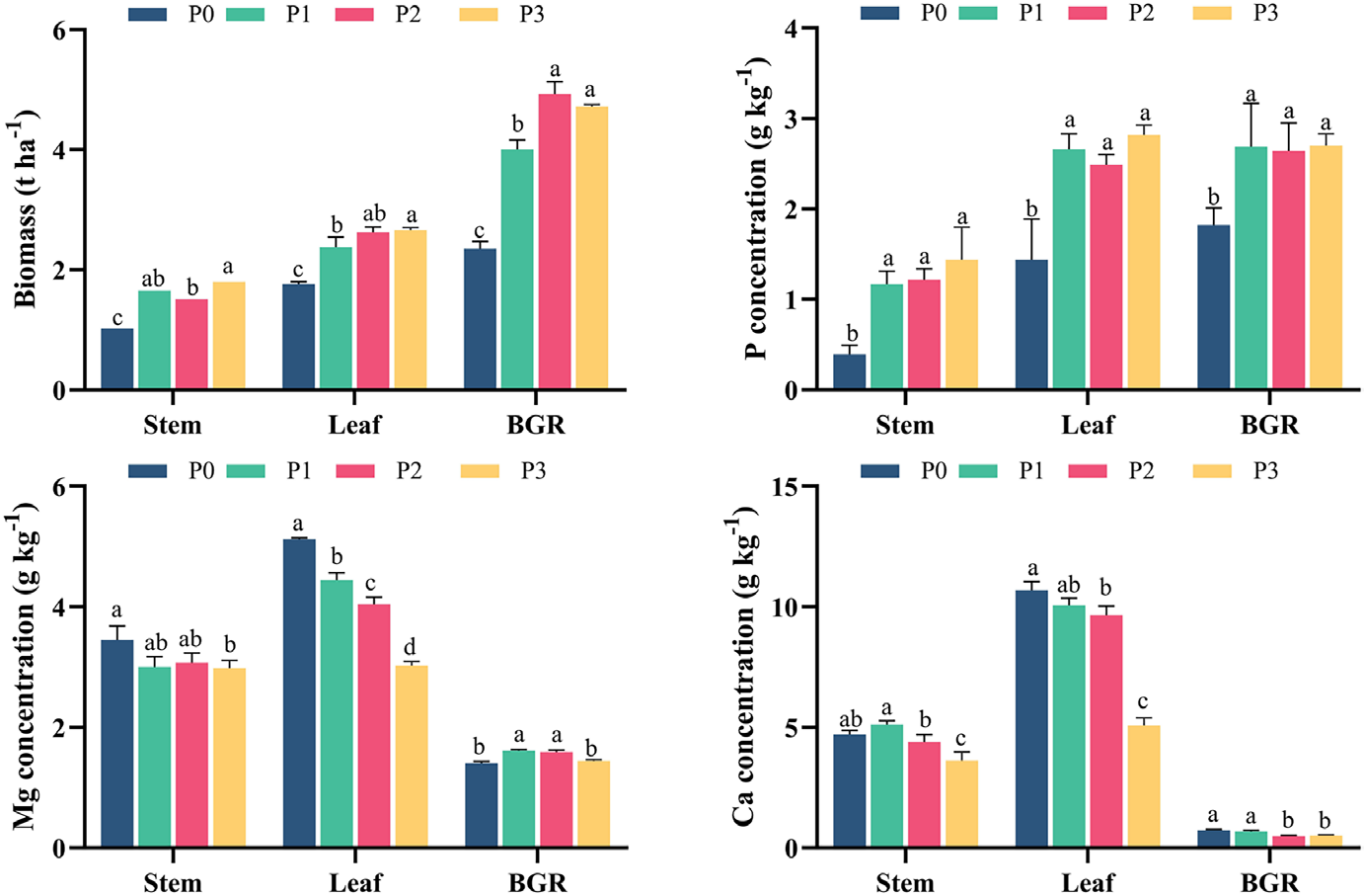
Influence of phosphorus (P) fertilizer application on the biomass and concentrations of phosphorus (P), magnesium (Mg) and calcium (Ca) in the stem, leaf, and BGR of sweet corn plant. Note: 1. BGR: bract+grain+rachis . 2. Bars are means of 3 replicates (*n* = 3). Different letters associated with bars in a specific plant organ indicate statistically significant differences at *p* < 0.05 (Tukey test). 3. P0 (0 kg P2O5 ha−1, no P supply), P1 (75 kg P2O5 ha−1, low P level), P2 (150 kg P2O5 ha−1, medium P level), and P3 (300 kg P2O5 ha−1, HP level).

The different P levels showed significant effects on biomass and the concentrations of Mg, Ca, and P in various above‐ground plant organs of sweet corn (Figure 1, Table S2). Mg and Ca concentrations were presented in the order of leaf > stem > BGR. By contrast, P concentration was displayed as leaf (BGR) > stem. On an average of four P levels, the Mg concentration in the leaf was 1.33 times higher than that in the stem (3.13 g kg^−1^) and 2.75 times higher than that in BGR (1.51 g kg^−1^), respectively. Conversely, the Ca concentration in the leaf (8.87 g kg^−1^) was approximately two times higher than that in the stem (4.46 g kg − 1) and 15 times higher than that in BGR (0.60 g kg − 1), respectively. The P concentration in the leaf (2.35 g kg^−1^) and BGR (2.46 g kg^−1^) was more than two‐fold higher than that in the stem (1.05 g kg^−1^).

P application increased biomass and P concentrations as expected and influenced Mg and Ca concentrations in various above‐ground organs (Table S2). For P concentration, stem P concentration had the highest coefficient of variation (CV = 42.9%) among different P levels, whereas BGR P had the lowest one (CV = 19.1%). Specifically, P concentrations in all three above‐ground plant organs were significantly higher with P application than with no P treatment (Figure 1). Mg concentration in the leaf (CV = 19.3%) had a larger change than those in the stem (CV = 8.0%) and BGR (CV = 6.40%) under different P levels (Table S2). Specifically, Mg concentrations in both the stem and leaf were significantly lower at HP levels compared to P_0–1_ levels (Figure 1). Among these, the leaf Mg concentration progressively decreased from 5.12 (P_0_) to 3.02 g kg^−1^ (P_3_) with an increased P level. In contrast, Mg concentration in BGR initially increased and then decreased, reaching significantly higher levels in P_1–2_‐treated plants compared to no P (P_0_) or the highest P level (P_3_) (Figure 1). For Ca concentration among different P levels, leaf Ca concentration had a larger change (CV = 26.3%) than those in the stem (CV = 13.8%) and BGR (CV = 19.8%) (Table S2). Comparatively, the Ca concentration in all three organs was more sensitive to P application than the Mg concentration, according to their respective CVs under P application (Table S2). Specifically, Ca concentrations in all three above‐ground plant parts were significantly lower in high‐P treatments than in P_0–1_ treatments. Compared to Ca concentrations under P_0_, Ca concentrations under P3 decreased by 23.1%, 52.4%, and 31.5% in the stem, leaf, and BGR, respectively (Figure 1).

### 3.3 Response of P Supply to Grain PAP, Mg, and Ca Concentrations and Their Bioavailabilities During the Grain Growth Stages

**FIGURE 2:**
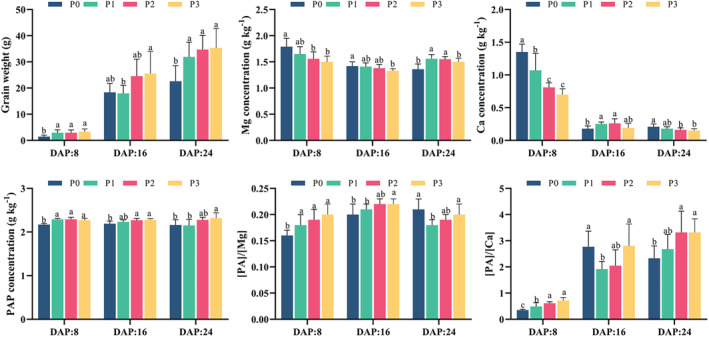
Effect of phosphorus (P) application on grain weight, magnesium (Mg) and calcium (Ca) concentrations, and their estimated bioavailabilities at different growth stages of sweet corn grain. Note: 1. DAP: days after pollination; PAP: phytic acid phosphorus; [PA]/[Mg]: the molar ratio of phytic acid to magnesium. [PA]/[Ca]: the molar ratio of phytic acid to calcium. 2. P0 (0 kg P2O5 ha−1, no P supply), P1 (75 kg P2O5 ha−1, low P level), P2 (150 kg P2O5 ha−1, medium P level), and P3 (300 kg P2O5 ha−1, HP level). 3. Bars are means of 3 replicates (*n* = 3). Different letters associated with bars in a specific grain growth stage indicate statistically significant differences at *p* < 0.05 (Tukey test).

At the whole cob scale, P application significantly influenced grain weight, PAP, Ca, and Mg concentrations, as well as their bioavailabilities, depending on the grain growth stage (Figure 2). P application significantly increased grain weight and grain PAP concentration at all grain growth stages. However, the effects of P application on grain Mg and Ca concentrations varied depending on the grain growth stage. At DAP 8 and 16, grain Mg concentration was significantly lower under the HP level (P_3_)‐treated sweet corn plant than under those treated with P_0–1_. In contrast, at DAP 24, P application (P_1–2_) significantly increased grain Mg concentration, which then decreased significantly at the P_3_ level. Ca concentration in P_1–2_‐treated grain significantly increased at DAP 16, while it decreased with increasing P levels at DAP 8 and 24.

For the grain bioavailabilities of Mg and Ca, a HP level (P_3_) increased the molar ratio of PA to Mg ([PA]/[Mg]) at DAP 8 and 16, but exhibited an initial decrease followed by an increase (lowest [PA]/[Mg] under P_1_ level) at DAP 24. HP increased grain [PA]/[Ca] at DAP 8 and 24, with an initial decrease followed by an increase at DAP 16. Those results revealed that HP (P_3_) significantly decreased the concentration of grain Mg and Ca and their bioavailabilities (a higher molar ratio indicated lower bioavailability), regardless of the grain growth stage (except grain Ca concentration in DAP 16). For harvested sweet corn (DAP 24), lower P levels favored higher bioavailabilities of Mg and Ca, with the highest Mg bioavailability in P_1_ and the highest Ca bioavailability in P_0–1_.

Based on the P‐induced CV values of quality parameters among different grain growth stages (Table S3), the highest CV value was obtained at DAP 8 for grain weight, Mg and Ca concentrations, and [PA]/[Mg]. By contrast, those for [PA]/[Ca] were observed at DAP 8 and 16, whereas the highest CV value for PAP concentration was obtained at DAP 24. This result showed that the alteration of grain weight and mineral nutrition induced by P treatment started as early as DAP 8. Grain weight, Mg and Ca concentrations, and their bioavailabilities were more sensitive to P supply at the early growth stage (DAP 8), while changes in PAP concentration were more prominent at the later growth stage (DAP 24, harvested stage).

### 3.4 Influence of P Application on Cob‐Position‐Dependent Grain PAP, Mg, and Ca Concentrations and Their Bioavailabilities During Grain Growth Stages

**FIGURE 3:**
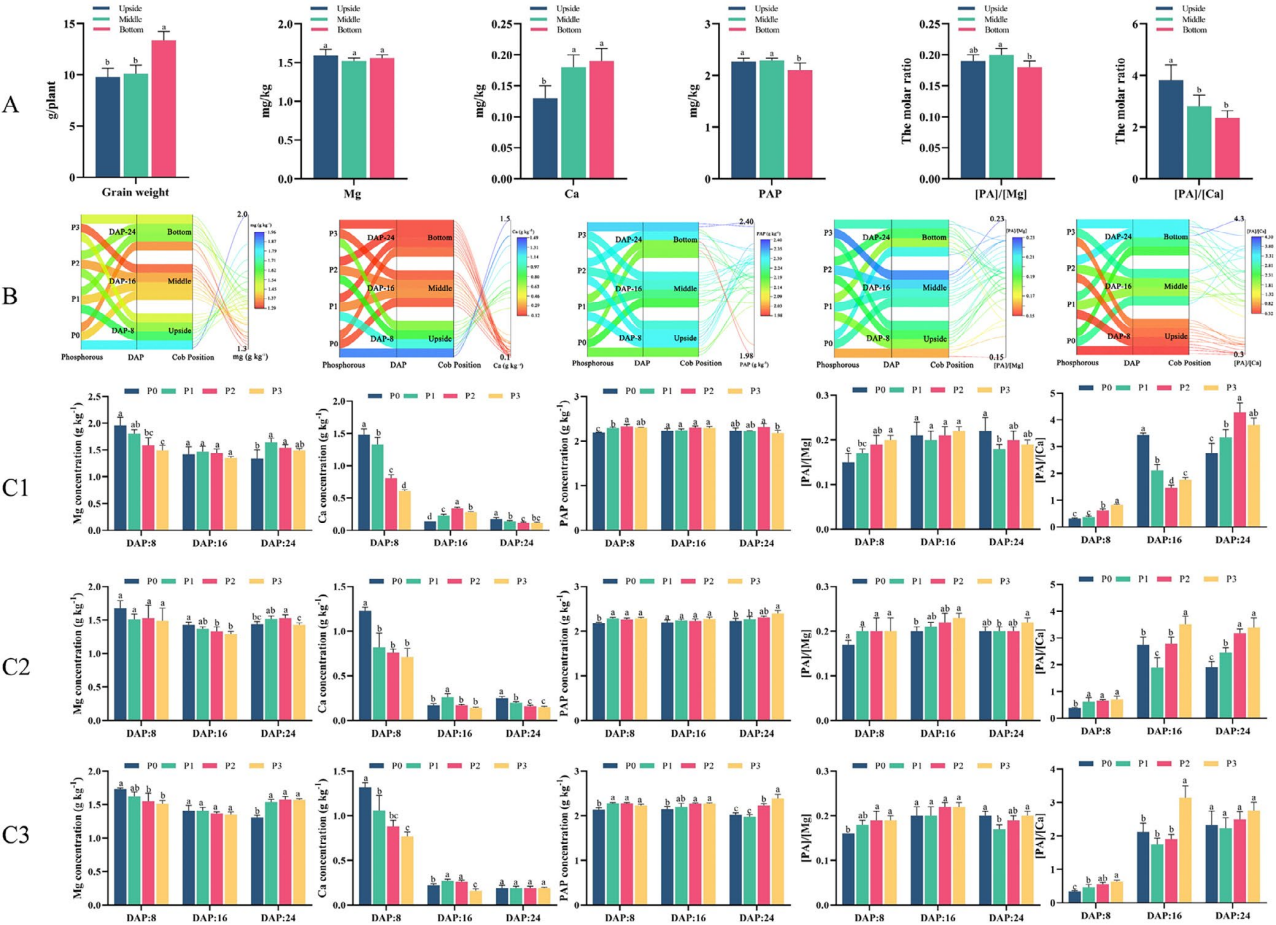
The distribution of grain weight, phytic acid phosphorus (PAP), and magnesium (Mg) and calcium (Ca) concentrations, as well as the minerals' estimated bioavailabilities at various positions on the cob, along with their dynamic response to application of phosphorus (P) in sweet corn. Note: 1. A: The distribution of grain weight, PAP, Mg, and Ca concentrations, as well as mineral’s estimated bioavailabilities in different parts of the cob (upside, middle, and bottom). 2. B: A parallel plot illustrating the interaction of quality parameters under varying levels of P, at different grain growth stages, and in different locations on the cob (upside, middle, and bottom). 3. P0 (0 kg P2O5 ha−1, no P supply), P1 (75 kg P2O5 ha−1, low P level), P2 (150 kg P2O5 ha−1, medium P level), and P3 (300 kg P2O5 ha−1, HP level). 4. C1: Effect of P application on grain weight, PAP, Mg, Ca concentration and their estimated bioavailabilities at various grain growth stage (DAP:8, 16, 24) in the upside of the cob. 5. C2: Effect of P application on grain weight, PAP, Mg, Ca concentration and their estimated bioavailabilities at various grain growth stage (DAP:8, 16, 24) in the middle of the cob. 6. C3: Effect of P application on grain weight, PAP, Mg, Ca concentration and their estimated bioavailabilities at various grain growth stage (DAP:8, 16, 24) in the bottom of the cob. 7. The terms upside, middle, and bottom refer to the location of the grain at different positions on the cob. 8. [PA]/[Mg]: the molar ratio of phytic acid to magnesium. [PA]/[Ca]: the molar ratio of phytic acid to calcium; DAP: days after pollination. 9. Bars are means of 3 replicates (*n* = 3). Different letters associated with bars in a specific grain growth stage indicate statistically significant differences at *p* < 0.05 (Tukey test).

Significant differences in grain weight, PAP, Mg, and Ca concentrations, as well as mineral bioavailabilities, were observed among different cob positions (Figure 3). The grain weight (g plant^−1^, on a dry matter basis) located at the bottom was significantly higher than at the upside and middle positions. Grain Ca concentrations at the middle and bottom positions were significantly higher than at the upside. However, there was no positional difference in Mg concentration among different cob positions. Conversely, the grain PAP concentration was significantly lower at the bottom position. Regarding the mineral bioavailabilities, the [PA]/[Mg] and [PA]/[Ca] at the bottom were significantly lower than those at the upside and/or middle positions, suggesting relatively higher Mg and Ca bioavailabilities in the bottom parts of the cob. Specifically, the grain weight at the bottom was 1.36 times higher than that at the upside position. Compared with grains located at the upside, the Ca concentration in grains at the bottom increased by 48.7%. By contrast, the PAP concentration, [PA]/[Mg], and [PA]/[Ca] decreased by 7.2%, 5.5%, and 38.1%, respectively.

P fertilizer influenced grain PAP, Mg, and Ca concentrations, as well as mineral bioavailabilities, depending on grain growth stage and cob position. For the grains located at the upside and bottom of the cob, a higher P level significantly decreased grain Mg concentrations at DAP 8, showed no variation at DAP 16, and significantly increased at DAP 24. By contrast, for the grains located in the middle of the cob, a higher P level resulted in an insignificant variation at DAP 8, a significant decrease at DAP 16, and an initial increase followed by a decrease at DAP 24. P‐induced variations in grain Ca concentrations depended on grain growth stage but were generally independent of cob position. Specifically, as the P level increased, the grain Ca concentration at all cob positions showed a progressive decrease at DAP 8. This was followed by an initial increase and subsequent decrease at DAP 16, and a significant decrease at DAP 24 (except for the Ca concentration in the bottom of the cob at DAP 24). P supply also significantly impacted grain PAP concentration, with HP generally resulting in higher PAP (except in the upside and middle of the cob at DAP 16 and upside at DAP 24). P supply increased PAP concentration across all cob positions at the early grain growth stage (DAP 8). At DAP 16, a significant increase in grain PAP concentration was only observed at the bottom of the cob. By DAP 24, P supply consistently increased grain PAP concentration in the middle and bottom of the cob, but not at the upside. The effect of P supply on grain [PA]/[Mg] and [PA]/[Ca] was generally opposite to its effect on grain Mg and Ca concentrations. Higher P levels led to a significant increase in [PA]/[Mg] at DAP 8 (except for middle‐cob grain) and 16 (except for upside‐cob grain), regardless of grain position in the cob. At DAP 24, [PA]/[Mg] initially decreased and then increased in the upside and bottom cob grains, with a general increase in middle‐cob grains. For [PA]/[Ca], higher P levels led to a significant increase at DAP 8 and 24, regardless of grain position on the cob (except for grains on the bottom at DAP 24). By contrast, an initial decrease was followed by an increase in upside and middle‐cob grains and a general increase in bottom cob grains at DAP 16.

To better understand the extent of P's influence on grain quality, P‐induced CV values were further analyzed (Tables S4 and S5). From a cob‐position perspective, the highest P‐induced CV values for PAP concentration were observed in grains at the bottom of the cob, whereas those for Mg and Ca concentrations and their bioavailabilities were at the upside of the cob (Table S4). Taking into account the grain cob position and the grain growth stage, the highest P‐induced CV values for PAP concentration were observed in grains located at the bottom of the cob at DAP 24. Conversely, the highest values for Mg and Ca concentrations, as well as their bioavailabilities ([PA]/[Mg] and [PA]/[Ca]), were observed in grains at the upside of the cob at DAP 8 (Table S5).

### 3.5 Influence of P Application on Grain Fraction‐Dependent Grain PAP, Mg, and Ca Concentrations and Their Bioavailabilities in Sweet Corn Grain

**FIGURE 4:**
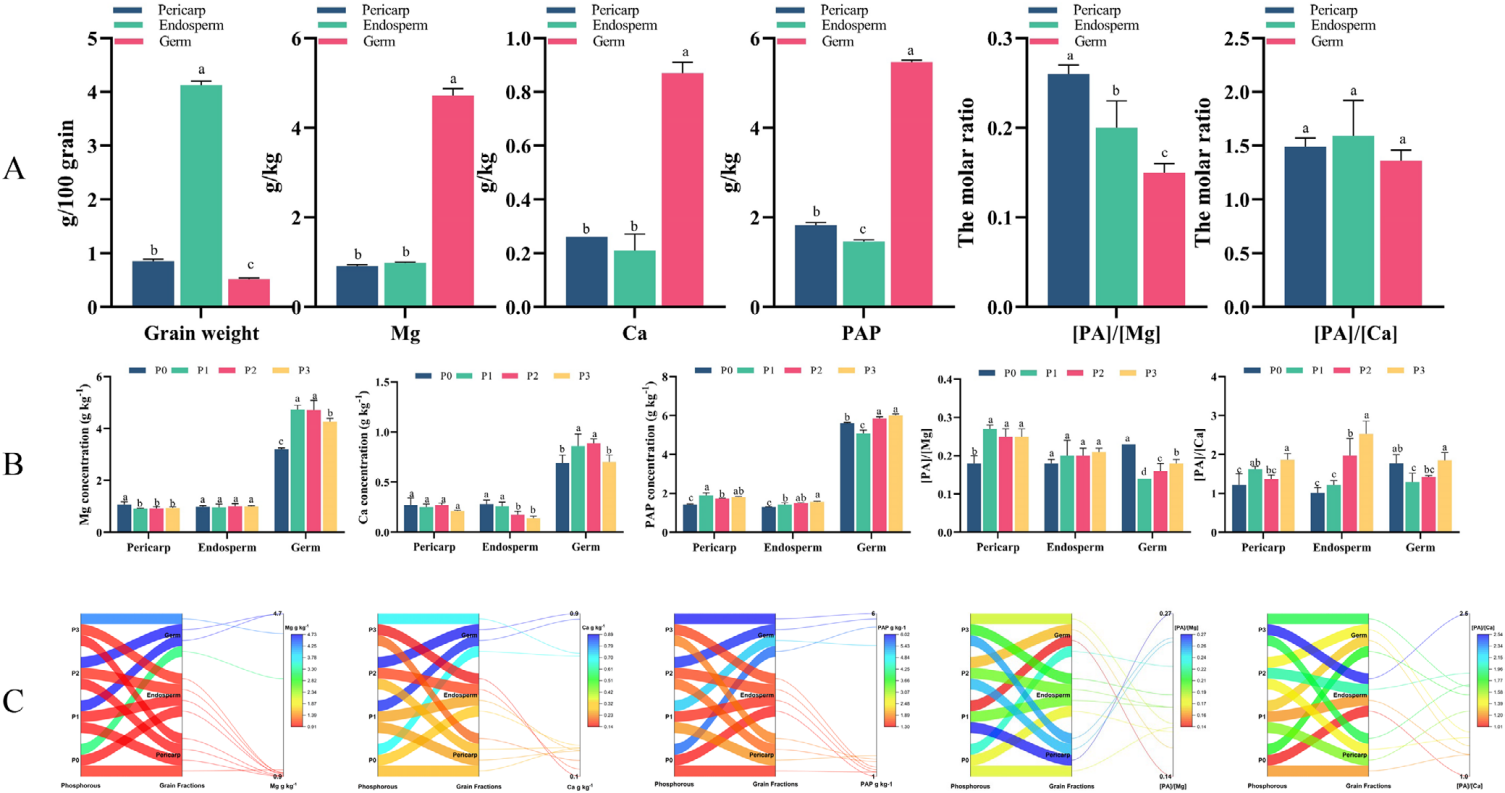
The distribution of grain weight, phytic acid phosphorus (PAP), and magnesium (Mg) and calcium (Ca) concentrations, as well as the minerals' estimated bioavailabilities at various grain fractions and their response to the application of phosphorus (P) in sweet corn grain. Note: A: The distribution of grain weight, PAP, Mg, and Ca concentrations, as well as mineral’s estimated bioavailabilities in different grain fraction (pericarp, endosperm and germ). B: A parallel plot illustrating the interaction of quality parameters under varying levels of P and in different grain fraction (pericarp, endosperm and germ). C: Effect of P application on grain weight, PAP, Mg, Ca concentrations, as well as the mineral’s estimated bioavailability at various grain fractions[PA]/[Mg]: the molar ratio of phytic acid to magnesium. [PA]/[Ca]: the molar ratio of phytic acid to calcium.P0 (0 kg P2O5 ha−1, no P supply), P1 (75 kg P2O5 ha−1, low P level), P2 (150 kg P2O5 ha−1, medium P level), and P3 (300 kg P2O5 ha−1, HP level)Bars are means of 3 replicates (*n* = 3). Different letters associated with bars in a specific grain fraction indicate statistically significant differences at *p* < 0.05 (Tukey test).

An uneven distribution of quality parameters was observed in harvested sweet corn grains (Figure 4). As expected, the endosperm contributed the most to the total dry weight of the grain, while the germ had the lowest weight and proportion. The concentrations of grain Mg (4.23 g kg^−1^), Ca (0.87 g kg^−1^), and PAP (5.64 g kg^−1^) in the germ were significantly higher than those in endosperm and pericarp fractions. PAP concentration in the pericarp (1.82 g kg^−1^) was significantly higher than that of the endosperm (1.46 g kg^−1^). By contrast no significant difference was observed between the pericarp and endosperm for Mg and Ca concentrations. On average, Mg concentrations in the germ were almost five times higher than those in the pericarp (5.2‐fold) and endosperm (4.8‐fold). Similarly, Ca concentrations in the germ were three to four times higher than those in the pericarp (3.3‐fold) and endosperm (4.1‐fold), and PAP concentrations in the germ were almost 3 times higher than those in the pericarp (3.0‐fold) and endosperm (3.7‐fold). Despite the significantly higher mineral concentrations in the germ, the molar ratio of PA to minerals showed a different pattern. The [PA]/[Mg] ratio was highest in the pericarp, followed by the endosperm and germ, with the pericarp having 1.3‐ and 1.7‐times higher [PA]/[Mg] than the endosperm and germ, respectively. However, no significant difference in [PA]/[Ca] was observed among various grain fractions.

P application significantly influenced the quality parameters of various grain fractions but did not alter the overall distribution pattern of Mg, Ca, PAP, and [PA]/[Ca], except for [PA]/[Mg]. Specifically, the influence of P application on grain PAP, minerals, and their bioavailabilities was dependent on the grain pearling fraction, with the magnitude of change varying from fraction to fraction. As P supply levels increased, Mg concentrations decreased in the pericarp and initially increased, then decreased in the germ, whereas no significant variation was observed in the endosperm. For Ca concentration, there was no significant variation in the pericarp with increasing P levels. However, there was a noticeable decrease in the endosperm from P_0–1_ to P_2–3_ and an initial increase followed by a decrease in the germ. The highest Ca concentration in the germ was observed at P_1–2_ levels. HP induced significantly higher PAP concentrations in all three fractions than no P‐treated (pericarp, endosperm) or low P_1_‐treated (germ) levels. P supply also significantly influenced the molar ratio of PA to minerals in different grain pearling fractions. Higher P levels initially decreased, then increased [PA]/[Mg] and [PA]/[Ca] in the germ, and significantly increased them in the pericarp. HP also led to a higher [PA]/[Ca] but was insignificant for [PA]/[Mg] in the endosperm.

The P‐induced CV among various grain fractions revealed that the highest CV for PAP concentration was observed in the pericarp. The endosperm exhibited the highest P‐induced CV for Ca and its bioavailability ([PA]/[Ca]), whereas the germ showed the highest P‐induced CV for Mg and its bioavailability ([PA]/[Mg]) (Table S6).

## 4 Discussion

Our results demonstrated the potential favorable role of P fertilization in increasing yield (Figures 1 and 2). However, a trade‐off between crop yield and mineral nutrition performance occurred when addressing P fertilization.

### 4.1 Status of Grain Mg, Ca, and PAP Concentrations and Minerals' Availability in Sweet Corn

Grain Mg and Ca concentrations in sweet corn were generally comparable to or lower than those found in other cereals (Broadley and White 2010; Gashu et al. 2021; Gu et al. 2015). In the UK, the legislation requires processed wheat flour to be mineral fortified, and the flour Ca concentration should be within the range of 2.35–3.90 g kg^−1^ (Broadley and White 2010), which is more than 15 times higher than the Ca concentration in our sweet corn grain. The challenge of “hidden hunger” is further complicated by the fact that mineral absorption in the human body depends not only on the quantity of minerals but also on the quantity of antinutritional factors such as PA (*myo*‐inositol‐1,2,3,4,5,6‐hexa*kis* phosphoric acid, C_6_H_18_O_24_P_6_). PA emerges as the predominant P compound in cereal grains. PAP is the primary way P is stored in cereal plant grains. In this study, we found that grain PA concentration among four sweet corn genotypes ranged from 7.38 to 8.30 g kg^−1^ across two P levels (Table 1), representing relative lower levels of PAP concentration than that among common maize and most other cereals (Schlemmer et al. 2009; Gu et al. 2015; Gashu et al. 2021). PA has long been considered an antinutritional compound, having a detrimental effect on mineral absorption in the human body. Liu et al. (2023) showed that about one‐third of the dietary Ca^2+^ in the gastrointestinal tract was chelated by PA, forming a Ca^2+^‐PA complex. Using stable isotopes, Bohn et al. (2004) found that the addition of PA to white‐wheat bread decreased fractional apparent Mg absorption from 32.5% (no added PA) to 13.0% (added 1.49 mmol PA) in humans. Those results reveal the close interaction between PA and the bioavailabilities of Mg and Ca. Here, we used the molar ratio of PA to minerals as a common indicator for evaluating mineral bioavailability (Su et al. 2018). The commonly assumed critical molar ratio values for the risk of bioavailability decrement are 0.24 for [PA]/[Ca] and 0.2 for [PA]/[Mg] (Rivera‐Martin, Broadley, and Poblaciones 2020). Our results suggested that although the bioavailabilities of Mg and Ca in sweet corn being superior to those in common maize grains, they still cannot be considered a food source with high Ca bioavailability. However, sweet corn shows greater potential for Mg availability, which is more promising compared to Ca availability.

### 4.2 Spatial Distribution of Grain PAP, Mg, and Ca Concentrations and Minerals' Availability in Sweet Corn

The nutrient concentration of cereal grains is significantly influenced by the grain's spatial distribution. At the spike scale in rice, Iwai et al. (2012) found that superior grains had significantly higher concentrations of most minerals compared to inferior grains. However, Melo‐Durán et al. (2021) indicated that the distributional pattern varies at the cob scale; grains located at the apical side possess less protein and starch but higher total/soluble nonstarch polysaccharides and fibers. Our results revealed that higher grain Ca concentrations were observed at the middle and bottom of the cob (superior grains). However, Mg concentration in harvested grains did not significantly differ among cob positions (Figure 3). These results demonstrate that, in addition to the possible nutrient flow distance and heavy‐grain‐induced dilution effect, other factors such as genotypes, growth stage, or harvested time should be considered when analyzing nutrient distribution in the cob/spike scale of cereal grains. In contrast to Mg and Ca, we found that inferior grains at the top of the cob had significantly higher PAP concentrations, whereas superior grains at the middle and bottom had lower PAP levels. Research has found that sucrose inhibits the expression of the l‐myo‐inositol‐1‐phosphate synthase gene, a rate‐limiting gene in PA biosynthesis (Yoshida, Fujiwara, and Naito 2002). Thus, the relatively lower grain PAP concentration in the bottom superior grains may be attributed to the inhibitive effect of a sufficient assimilate supply, such as sucrose, on grain PA synthesis during grain filling. Furthermore, grains at the bottom of the cob had significantly higher Mg and Ca bioavailabilities.

Similar to the cob scale, the nutrient distribution in grain fractions was also uneven. Most minerals and nutritive components in grains accumulate in the aleurone layer and germ (Iwai et al. 2012; Su et al. 2018). Our results align with previous findings, revealing that the Mg, Ca, and PAP concentrations in the germ were approximately three to five‐fold higher than those in other grain fractions (Wang et al. 2011). However, the PA concentration in the pericarp of sweet corn was noticeably lower than that of wheat or rice bran (Gupta, Gangoliya, and Singh 2015). Furthermore, although the germ fraction possesses higher PAP and mineral (Mg and Ca) concentrations, mineral bioavailabilities were contrasting (Figure 4). The highest Mg bioavailability was in the germ fraction, followed by the endosperm and pericarp, whereas Ca bioavailability remained consistent across all grain fractions.

### 4.3 Influence of P Fertilization on Grain Mg, Ca, and PAP Concentrations and the Bioavailabilities of Mg and Ca in Sweet Corn

Studies in rice indicated that P application could promote an increase in grain Mg concentration (Rose et al. 2016). Conversely, other studies have observed an increase in grain Mg at low soil P levels, suggesting that reduced P uptake may lead to a higher proportion of Mg being partitioned to the grain (Penn, Camberato, and Wiethorn 2023). In our study on sweet corn, the effect of P on grain Mg concentration was dose‐dependent. Optimal P supply increased grain Mg concentration, but excessive P levels significantly reduced it (Figures 1 and 2). The detailed spatial–temporal data further indicated that, temporally, the early grain growth stage exhibited a higher sensitivity to P‐induced changes in grain Mg concentration. Spatially, optimal P application significantly increased harvested grain Mg concentration across all cob positions and in the Mg‐enriched germ fraction. However, when P levels were high, there was a significant decrease in harvested grain Mg concentration in the middle‐cob grains and germ fractions, which consequently influenced the whole harvested grain Mg concentration (Figures 3 and 4).

The effect of P on grain Ca concentrations differed from its impact on Mg concentration (Figures 2, 3, 4). We observed a substantial inhibitory effect of P on the Ca concentration across all three above‐ground organs (Table 1, Figures 1 and 2). HP application reduced grain Ca concentration by 28.6%, a reduction greater than that in Zn concentration (16.6% for wheat and 20.2% for maize) caused by P (Zhang et al. 2021). The detailed spatial–temporal analysis revealed that, temporally, P primarily influenced grain Ca concentration during the early grain growth stage, a pattern similar to that observed for grain Mg concentration. Spatially, HP levels induced a decrease in grain Ca concentration in the upside and middle parts of the cob, as well as in the germ and endosperm fractions. This is likely the cause of the overall decrease in harvested grain Ca concentration under HP conditions.

P application significantly increased grain PAP concentration in sweet corn (Table 1, Figure 2). This was consistent with other cereal plants such as rice and wheat, although with a relatively smaller increase (7.5%) (Su et al. 2018; Zhang et al. 2017, 2021). The detailed spatial–temporal data further indicated that HP consistently elevated grain PAP concentration across all growth stages, cob positions, and grain fractions, compared with no P and low P conditions. Moreover, the effect of P supply on grain mineral bioavailabilities ([PA]/[Mg] and [PA]/[Ca]) was typically inverse to its effect on grain Mg and Ca concentrations (Table 1, Figure 2). This suggests that the relatively lower grain Ca bioavailability was further decreased with P application. The spatial–temporal results revealed that, temporally, P primarily influenced grain PAP concentration during the late grain growth stage, whereas it influenced the bioavailabilities of grain Mg and Ca during the early grain growth stage. Spatially, HP consistently increased PAP concentration across all growth stages, all grain fractions, and the middle and bottom cob positions. However, it significantly reduced the bioavailabilities of grain Mg and Ca across all cob positions and grain fractions.

## 5 Conclusions

The early grain growth stage was more sensitive to P‐induced changes in grain Mg and Ca concentrations and their bioavailabilities, whereas the late stage was influenced by P in terms of grain PAP concentration. P application progressively decreased grain Ca concentration, primarily due to reductions in the upper and middle parts of the cob, as well as in the germ and endosperm fractions. In contrast, P application consistently increased grain PAP concentration but decreased grain Mg and Ca bioavailabilities across all grain growth stages and grain fractions. Optimal P levels increased Mg concentration across all cob positions and in the germ fraction, while high P (HP) levels decreased Mg concentration, especially in middle‐cob grains and germ fractions.

## Author Contributions

**Da Su:** funding acquisition (equal), methodology (equal), writing – original draft (equal). **Zhiya Jin:** data curation (equal), validation (equal). **Jie Ou:** data curation (equal), validation (equal). **Muhammad Atif Muneer:** validation (equal), writing – review and editing (equal). **Yunfei Jiang:** writing – review and editing (equal). **Delian Ye:** writing – review and editing (equal). **Liangquan Wu:** writing – review and editing (equal). **Xiaojun Yan:** conceptualization (equal), data curation (equal), methodology (equal), resources (equal), writing – review and editing (equal).

## Conflicts of Interest

The authors declare no conflicts of interest.

## Supporting information


Table S1.

## Data Availability

Data available on request from the authors.
