# Supplementary material for: Spatial–Temporal Variation of Grain Magnesium, Calcium and Their Response to Phosphorus Nutrition in Sweet Corn
Source: Food Sci Nutr. 2025 Jan 25;13(1):e4725. doi: 10.1002/fsn3.4725 (PMC11762451; doi:10.1002/fsn3.4725)
Supplement: Supplementary file 1 — Table S1. [file FSN3-13-e4725-s001.docx]

**Supplementary Information**

**Spatial–temporal variation of grain magnesium, calcium and their response to phosphorus nutrition in sweet corn**

Da Su ^1, 2^, Zhiya Jin ^1^, Jie Ou ^2^, Muhammad Atif Muneer ^2^, Yunfei Jiang ^3^, Delian Ye ^1, 2^, Liangquan Wu ^2^, Xiaojun Yan ^2^

**Affiliations:**

^1^ Key Laboratory of Genetics, Breeding and Multiple Utilization of Crops, Ministry of Education; Key Laboratory of Biological Breeding for Fujian and Taiwan Crops, Ministry of Agriculture and Rural Affairs, College of Agriculture, Fujian Agriculture and Forestry University, Fuzhou, 350002, China

^2^ International Magnesium Institute, College of Resources and Environment, Fujian Agriculture and Forestry University, Fuzhou, 350002, China

^3^ Department of Plant, Food, and Environmental Sciences, Faculty of Agriculture, Dalhousie University, Truro, NS, B2N 5E3, Canada

**^*^ Corresponding author**: 16565710@fafu.edu.cn

**In total 7 pages, 6 tables.**

Table S1: The basic soil characteristics.

|  | PH | Organic C (g kg^-1^) | Total N  (g kg^-1^) | Available P (g kg^-1^) | Available K (g kg^-1^) | Exchangeable Mg (g kg^-1^) | Exchangeable Ca (g kg^-1^) |
| --- | --- | --- | --- | --- | --- | --- | --- |
| 2018 | 5.89 | 5.64 | 0.76 | 0.0058 | 0.0551 | 0.0518 | 0.2546 |
| 2019 | 5.90 | 5.20 | 0.60 | 0.0082 | 0.0402 | 0.0524 | 0.2699 |

Table S2: The mean and coefficient of variation (CV, %) of phosphorus-induced variations in biomass, and the concentrations of phosphorus (P), magnesium (Mg), and calcium (Ca) in the stem, leaf, and BGR of the sweet corn plant across four P levels

|  | Biomass (t ha^-1^) | | |  | P (g kg^-1^) | | |  | Mg (g kg^-1^) | | |  | Ca (g kg^-1^) | | |
| --- | --- | --- | --- | --- | --- | --- | --- | --- | --- | --- | --- | --- | --- | --- | --- |
|  | stem | leaf | BGR |  | stem | leaf | BGR |  | stem | leaf | BGR |  | stem | leaf | BGR |
| Mean | 1.49 | 2.36 | 4.00 |  | 1.05 | 2.35 | 2.46 |  | 3.13 | 4.15 | 1.51 |  | 4.46 | 8.87 | 0.60 |
| CV | 21.0 | 16.3 | 26.6 |  | 42.9 | 25.69 | 19.1 |  | 8.0 | 19.3 | 6.4 |  | 13.8 | 26.3 | 19.8 |

Note:

1. BGR: bract+grain+rachis
2. Mean: the mean value of four P treatments

Table S3: Mean values and coefficients of variation (CV, %) of phosphorus-induced changes in grain weight, concentrations of magnesium (Mg) and calcium (Ca), and their estimated bioavailabilities at different grain growth stages of sweet corn.

|  | Grain weight (g) | | |  | PAP (g kg^-1^) | | |  | Mg (g kg^-1^) | | |  | Ca (g kg^-1^) | | |  | [PA]/[Mg] | | |  | [PA]/[Ca] | | |
| --- | --- | --- | --- | --- | --- | --- | --- | --- | --- | --- | --- | --- | --- | --- | --- | --- | --- | --- | --- | --- | --- | --- | --- |
|  | DAF 8 | DAF 16 | DAF 24 |  | DAF 8 | DAF 16 | DAF 24 |  | DAF 8 | DAF 16 | DAF 24 |  | DAF 8 | DAF 16 | DAF 24 |  | DAF 8 | DAF 16 | DAF 24 |  | DAF 8 | DAF 16 | DAF 24 |
| Mean | 0.89 | 7.20 | 10.38 |  | 2.26 | 2.24 | 2.23 |  | 1.62 | 1.39 | 1.49 |  | 0.98 | 0.22 | 0.17 |  | 0.18 | 0.21 | 0.20 |  | 0.54 | 2.38 | 2.91 |
| CV | 44.23 | 30.45 | 25.16 |  | 2.55 | 1.92 | 5.69 |  | 9.01 | 3.75 | 6.53 |  | 29.70 | 29.05 | 21.70 |  | 9.99 | 5.28 | 7.75 |  | 29.48 | 29.69 | 24.16 |

Note:

1. PAP (phytic acid phosphorus); Mg (magnesium); Ca (calcium); [PA]/[Mg]: the molar ratio of phytic acid to magnesium; [PA]/[Ca]: the molar ratio of phytic acid to calcium; DAF: days after flowering.
2. Mean: the mean value of four P treatments.

Table S4: Mean values and coefficients of variation (CV, %) of phosphorus-induced changes in grain weight, concentrations of magnesium (Mg) and calcium (Ca), and their estimated bioavailabilities at different cob positions throughout the entire growth stages of sweet corn

|  | PAP | | Mg | | Ca | | [PA]/[Mg] | | [PA]/[Ca] | |
| --- | --- | --- | --- | --- | --- | --- | --- | --- | --- | --- |
|  | Mean  (g kg^-1^) | CV  (%) | Mean  (g kg^-1^) | CV  (%) | Mean  (g kg^-1^) | CV  (%) | Mean | CV  (%) | Mean | CV  (%) |
| Upside | 2.26 | 2.27 | 1.54 | 11.88 | 0.48 | 100.05 | 0.19 | 11.38 | 2.09 | 67.58 |
| Middle | 2.27 | 2.50 | 1.46 | 7.18 | 0.42 | 86.83 | 0.20 | 7.85 | 2.02 | 57.89 |
| Bottom | 2.20 | 5.20 | 1.50 | 8.47 | 0.48 | 87.23 | 0.20 | 10.30 | 1.73 | 56.59 |

Note:

PAP (phytic acid phosphorus); Mg (magnesium); Ca (calcium); [PA]/[Mg]: the molar ratio of phytic acid to magnesium; [PA]/[Ca]: the molar ratio of phytic acid to calcium.

Mean: the mean value of all four treatments

Table S5: Coefficient of variation (CV, %) for nutritive components at different phosphorus (P) levels across various cob position and grain growth stages.

| Cob-position |  | DAF 8 | DAF 16 | DAF 24 |
| --- | --- | --- | --- | --- |
| Upside | PAP (g kg^-1^) | 2.72 | 1.43 | 2.64 |
|  | Mg (g kg^-1^) | 12.30 | 3.52 | 8.35 |
|  | Ca (g kg^-1^) | 39.43 | 34.18 | 19.25 |
|  | [PA]/[Mg] | 14.50 | 3.98 | 9.98 |
|  | [PA]/[Ca] | 43.23 | 39.72 | 18.52 |
| Middle | PAP (g kg^-1^) | 2.24 | 1.40 | 3.11 |
|  | Mg (g kg^-1^) | 5.44 | 4.54 | 3.35 |
|  | Ca (g kg^-1^) | 27.15 | 27.51 | 24.51 |
|  | [PA]/[Mg] | 7.71 | 6.41 | 5.23 |
|  | [PA]/[Ca] | 24.41 | 24.19 | 24.89 |
| Bottom | PAP (g kg^-1^) | 2.92 | 2.70 | 8.75 |
|  | Mg (g kg^-1^) | 6.07 | 2.07 | 8.50 |
|  | Ca (g kg^-1^) | 23.96 | 22.81 | 1.67 |
|  | [PA]/[Mg] | 7.87 | 5.81 | 7.53 |
|  | [PA]/[Ca] | 23.87 | 28.27 | 9.52 |

Note:

PAP (phytic acid phosphorus); Mg (magnesium); Ca (calcium); [PA]/[Mg]: the molar ratio of phytic acid to magnesium; [PA]/[Ca]: the molar ratio of phytic acid to calcium; DAF: days after flowering.

Table S6: Coefficients of variation (CV, %) for nutritive components at different phosphorus (P) levels across different grain fractions

| Grain fraction | PAP (g kg^-1^) | Mg (g kg^-1^) | Ca (g kg^-1^) | [PA]/[Mg] | [PA]/[Ca] |
| --- | --- | --- | --- | --- | --- |
| Pericarp | 11.90 | 7.54 | 11.05 | 17.05 | 18.92 |
| Endosperm | 7.92 | 1.90 | 32.46 | 6.48 | 41.84 |
| Germ | 7.18 | 16.95 | 13.09 | 21.34 | 16.92 |

Note: PAP (phytic acid phosphorus); Mg (magnesium); Ca (calcium); [PA]/[Mg]: the molar ratio of phytic acid to magnesium; [PA]/[Ca]: the molar ratio of phytic acid to calcium.
